# A systematic review and meta-analysis of 271 PCDH19-variant individuals identifies psychiatric comorbidities, and association of seizure onset and disease severity

**DOI:** 10.1038/s41380-018-0066-9

**Published:** 2018-06-11

**Authors:** Kristy L Kolc, Lynette G Sadleir, Ingrid E Scheffer, Atma Ivancevic, Rachel Roberts, Duyen H Pham, Jozef Gecz

**Affiliations:** 1 Adelaide Medical School, The University of Adelaide, Adelaide, SA Australia; 2 Department of Paediatrics and Child Health, University of Otago, Wellington, New Zealand; 3 Departments of Medicine and Paediatrics, Austin Health and Royal Children’s Hospital and Florey Institute, The University of Melbourne, Melbourne, VIC Australia; 4 School of Psychology, The University of Adelaide, Adelaide, SA Australia; 5 Robinson Research Institute, The University of Adelaide, Adelaide, SA Australia; 6 Healthy Mothers and Babies, South Australian Health and Medical Research Institute, Adelaide, SA Australia

**Keywords:** Genetics, Autism spectrum disorders, Psychiatric disorders

## Abstract

Epilepsy and Mental Retardation Limited to Females (EFMR) is an infantile onset disorder characterized by clusters of seizures. EFMR is due to mutations in the X-chromosome gene *PCDH19*, and is underpinned by cellular mosaicism due to X-chromosome inactivation in females or somatic mutation in males. This review characterizes the neuropsychiatric profile of this disorder and examines the association of clinical and molecular factors with neuropsychiatric outcomes. Data were extracted from 38 peer-reviewed original articles including 271 individual cases. We found that seizure onset ≤12 months was significantly associated (*p* *=* 4.127 × 10^−7^) with more severe intellectual disability, compared with onset >12 months. We identified two recurrent variants p.Asn340Ser and p.Tyr366Leufs*10 occurring in 25 (20 unrelated) and 30 (11 unrelated) cases, respectively. *PCDH19* mutations were associated with psychiatric comorbidities in approximately 60% of females, 80% of affected mosaic males, and reported in nine hemizygous males. Hyperactive, autistic, and obsessive-compulsive features were most frequently reported. There were no genotype–phenotype associations in the individuals with recurrent variants or the group overall. Age at seizure onset can be used to provide more informative prognostic counseling.

## Introduction

Epilepsy and Mental Retardation Limited to Females (EFMR; OMIM #300088) was first described in 1971 by Juberg and Hellman as an early onset seizure disorder triggered by febrile illness, and with female-limited expression [1]. The causative gene was identified in 2008 by Dibbens et al. in a study that involved six new EFMR families, as well as the original EFMR family reported by Juberg and Helman [2]. In the same year, EFMR was further characterized as a neurological disorder with a markedly varied neuropsychiatric profile including intellectual disability (ID), and aggressive, autistic, or obsessive features [3]. In 2009, Depienne et al. identified *PCDH19* mutations in sporadic cases with infantile development and epileptic encephalopathy resembling Dravet Syndrome [4]. Males have since been identified who are cellular mosaics for the *PCDH19* gene with a similar clinical profile as that of affected females [4–6], thus challenging the dogma that this is a disorder limited exclusively to females. The hallmark feature of *PCDH19*-associated epilepsy is that seizures occur in clusters. We therefore proposed “girls clustering epilepsy” (PCDH19-GCE) as a name to facilitate clinical identification of this disorder [7]. Seizures typically present as generalized tonic-clonic and/or focal seizures, which may evolve to bilateral, tonic-clonic seizures. An additional unifying feature of PCDH19-GCE is cellular mosaicism, either due to X-chromosome inactivation in females or early somatic mutation and, as such, somatic mosaicism in males.

PCDH19-GCE is associated with a reduction or remission of seizures during adolescence [3, 8, 9]. Unfortunately, neuropsychiatric dysfunction remains, often exacerbating with age and becoming the most prominent and disabling feature in some patients [10–12]. ID ranging from mild to profound is present in approximately 70% of the cases [13]. The prevalence of psychiatric comorbidities is unknown, however, reports suggest that autism spectrum disorder (ASD) is a common feature in both females [14–16] and males [17]. Intriguingly, no association has been established between the severity of epilepsy and ID [9, 10]. Here we conduct a comprehensive and systematic review of the PCDH19-GCE literature, specifically focusing on the neuropsychiatric profile and examining whether associations exist between age at seizure onset, mutation type, variant location or mode of inheritance, and cognitive function or psychiatric comorbidity. Determining the factors that contribute to clinical outcome will be useful for prognostic counseling.

## Method

### Inclusion criteria

Studies were included if they met the following criteria: 1) reported the cDNA or protein change, 2) were peer-reviewed and written in English, and 3) were original cases only. For a study  to be included in the meta-analysis, information regarding cognitive function or the degree of impairment, or the presence or type of psychiatric comorbidity was also required.

### Search strategy

A computerized search of public databases Embase, PubMed, Google Scholar, and Scopus from January 2008 to August 2017 was conducted. The search terms were as follows: pcdh19, pcdh 19, protocadherin19, and protocadherin 19. Full-text articles of abstracts were then selected, retrieved, and assessed for eligibility considering the established criteria detailed above. Inclusion was based on final consensus between two authors. Authors were contacted via email if further information or clarification was required. The reference lists of all articles selected for review, and the full texts of the potentially relevant studies were also examined.

### Data extraction

All relevant data were extracted from selected articles and imported into a Statistical Program for the Social Sciences (SPSS) dataset. Data were cleaned and cross-checked to ensure that no individual was recorded more than once.

### Excluding duplicates

To minimize the bias through reporting of the same individual from multiple publications, we used the following information to identify duplicates: mutation (cDNA or protein change) and age at seizure onset. As additional measures, we also used the age at study and inheritance information to further confirm the likelihood that a case was the same across two or more publications. We annotated identified duplicates with a double asterisk (see Supplementary Raw Data). Once a potential duplicate was identified, the most recent duplicated information for that individual was included in the review and all references were assigned to that individual. For example, case 68 and 172 in the raw data were flagged as potential duplicates. Both cases were reported as having a *de novo* c.2097dupA mutation and an age at seizure onset of 7 months. In addition, the age of the individual when reported satisfied the expected change based on publication dates (11 months; 2010 and 2 years 2 months; 2012). If there was a slight discrepancy between suspected duplicates, caution was taken, and the suspected duplicate was removed. For example, case 110 and 187 in the raw data file were flagged as potential duplicates. Both cases were reported as having a c.1298T>C mutation, however with an age at seizure onset of 9 months and 7 months, respectively. As additional information such as age at study and inheritance satisfied the assumption of a duplicate, the case was only included once in this review.

### Data coding

Initially, certain variables were re-classified to aid the analyses. Seizure onset was classified as follows: (0) “*early*” (≤12 months) or (1) “*late*” (>12 months). Mutation type was classified as follows: (0) “*truncating*” or (1) “*missense*”. Other variants were too infrequent and, as such, were excluded from the analyses. Variant location was classified as (0) “*early*” (EC1 to EC3) or (1) “*late*” (EC4 to cytoplasmic), and inheritance as (0) “*sporadic*” or (1) “*familial*”. The first dependent variable (cognitive function) was scored on a scale: (0) “*normal*”, (1) “*borderline*”, (2) “*mild ID*”, (3) “*moderate ID*”, or (4) “*severe/profound ID*”, with higher scores indicating increased ID severity. Information regarding the degree of ID was extracted from reports only if explicitly stated and where a report indicated a range, i.e., moderate-to-severe ID, classification was based on the more severe category. Given that the number of levels of cognitive function exceeded four, this variable was upgraded to continuous [18]. The second dependent variable (psychiatric comorbidity) was scored as follows: (0) “*no psychiatric features reported*”, or (1) “*psychiatric features reported*”. Reports that did not cover this aspect of the clinical profile were coded “*N/A*” and excluded from the analyses.

### Missing data

Cognitive function was based on what was reported in the literature. Full-scale intelligence quotient scores were rarely reported. Generally, the reports involved reference to the classification of normal, borderline, mild ID, moderate ID, severe ID, or profound ID. As such, this classification was adopted for analytical purposes. There were some instances where the developmental quotients were provided. Although early developmental quotient testing had been shown to correlate with later IQ [19], they are not the same. Therefore, a classification could not be attributed to these cases and, as such, they were excluded from the analysis. If data were missing from any of the other variables in the meta-analysis, the case was excluded to prevent an over- or under-estimation of the true nature of any association. In total, 131 cases were represented in the meta-analysis.

### Data analyses

Continuous data were analyzed using SPSS version 24, and followed significant effects (*p* ≤ .05) using a linear regression model, factorial analysis of variance (ANOVA), and Student’s *t*-tests. For the binary categorical outcome variable, chi-squared tests of independence were performed. Descriptive statistics, scatterplots, and histograms were generated for all variables used in the analyses to ensure that the data met the criteria for the use of parametric tests. While the normality assumption within the levels of certain independent variables was not met, there were more than 30 cases in each group and, as such, the parametric tests could be utilized [20]. Furthermore, non-parametric tests yielded the same results for all parametric tests performed.

### PCDH19 reference sequences

All PCDH19 cDNAs and proteins were based on the following reference sequences, which represent the longest isoform of PCDH19 mRNA and protein: NM_001184880 and NP_001171809 (https://www.ncbi.nlm.nih.gov/).

## Results

Thirty-eight studies with a total of 297 cases met the inclusion criteria. After excluding the duplicates, there were a total of 271 individuals comprising 12 (4.4%) males and 259 (95.6%) females. Two males were excluded from the meta-analysis as they harbored hemizygous *PCDH19* mutations and exhibited a phenotype that was not characteristic of PCDH19-GCE. All ten mosaic males were included in all descriptive and statistical analyses, and did not differ significantly from females on the outcome measures tested (cognitive function: *t*_(193)_ *=* −0.33, *p* *=* 0.745; psychiatric comorbidity: *χ*^2^_(1, *n* = 230)_ = 1.99, *p* *=* 0.158). The mean age at time of the study (*n* = 235) was 13.0 years (SD = 12.1, range = 1–79 years). The average age at seizure onset (*n* = 219) was 11.9 months (SD = 9.0, median = 10 months, range = 1–70 months; see Supplementary Figure 1), with seizure onset precipitated by fever in 81.1% of cases where this information was available (see Supplementary Table 1).

### PCDH19 mutation

**Fig. 1:**
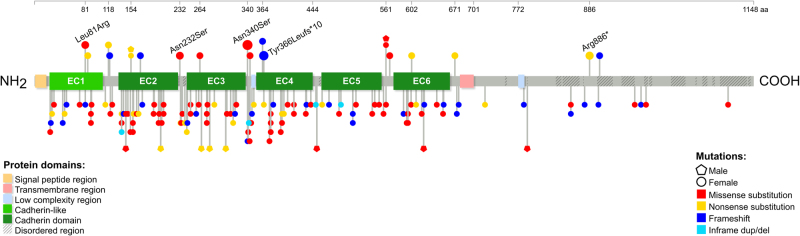
Lollipop plot illustrating all the reviewed PCDH19-GCE variants (*n* = 271). Lollipop size is exponentially proportional to the number of times the variant has been observed. Recurrent (i.e., seen more than once in unrelated individuals) variants are located above the protein and labeled if they occur more than twice

The *PCDH19* gene is located at Xq22.1 and its coding sequence consists of six exons. The gene encodes a 1148 amino acid protein with typical features of the δ2-protocadherin sub-family, with 23 amino acid signal peptides, six conserved cadherin repeats in the extracellular (EC) domain, a transmembrane domain, and conserved motifs (CM1-CM2) in the C-terminal region [2, 21]. The first exon encodes the extracellular and transmembrane domains, as well as a small portion of the C-terminal region. While the rest of the C-terminal region is encoded by exons 2–6, the second, and likely the third exon are subjected to alternative splicing. Exons 5 and 6 encode for CM1 and CM2, respectively. The majority of the reported PCDH19-GCE mutations were observed in the EC domain of the protein encoded by exon 1 (86.7%; Fig. 1). Of the reported variants in this region, almost half were located in the EC3 and EC4 domains (20.3% and 23.2%, respectively; see Supplementary Table 2). Missense variants were the most frequently reported type of PCDH19-GCE mutation (45.4%), followed by the frameshift (27.3%), and nonsense variants (19.6%; see Supplementary Table 3). In total, 145 unique germline *PCDH19* mutations were identified in PCDH19-GCE, both in large families as well as singleton cases (see Supplementary Table 4 for a complete list).

#### Mode of inheritance

PCDH19-GCE was originally recognized as a familial disorder [1, 3, 22]. However, in recent years a significant number of sporadic cases have been identified due to next-generation sequencing, with over half of reported PCDH19-GCE cases arising *de novo* (50.2%; Supplementary Table 5). Interestingly, there were a considerable number of maternally inherited mutations (18.7%). The penetrance of PCDH19-GCE has been estimated as greater than 90%, however, recent reports of asymptomatic carrier mothers [11, 12, 23] would suggest that this is somewhat lower. The difficulty in determining penetrance lies in the definition. Some studies define unaffected individuals by a complete absence of symptoms [10, 11], while others refer to an individual as being unaffected if they only had a brief history of infantile seizures [3, 11, 24]. Based on the inclusion of all reports where a mutation has been maternally inherited, we estimated the penetrance of PCDH19-GCE to be 80%. This may still be conservative, given that this number is based only on reported cases in which the mothers have been tested.

#### Recurrent variants

**Fig. 2:**
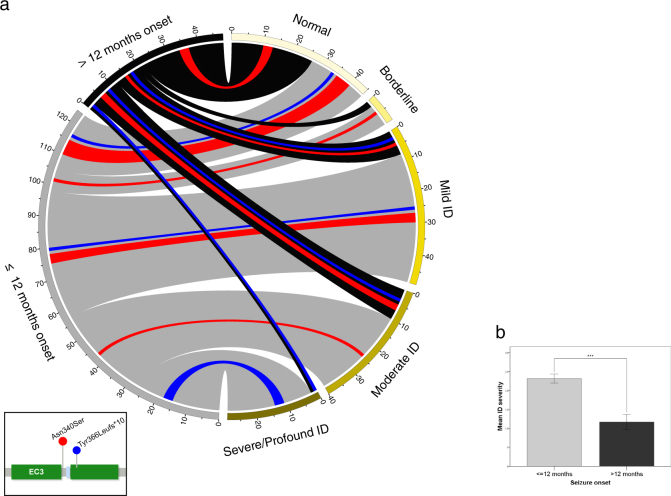
Genotype–phenotype association. a Circos plot illustrating the variable cognitive profile of PCDH19-GCE (*n* = 155) against age at seizure onset: ≤12 months (*n* = 124) and >12 months (*n* = 48). Recurrent variants p.Asn340Ser (*n* = 17) and p.Tyr366Leufs*10 (*n* = 23) are highlighted in red and blue, respectively. Axes show the number of individuals in each category. Illustration represents cases where relevant information was available. **b** Bar graph (±1 SEM) illustrating the association of age at seizure onset and ID severity (values from unadjusted linear model), ***p = 3.090 × 10^−7^. Cognitive function was scored on a scale: (0) “*normal*”, (1) “*borderline*”, (2), “*mild ID*”, (3) “*moderate ID*”, and (4) “*severe/profound ID*”, with higher scores indicating increased ID severity. Mean ID severity was derived by totaling the scores and dividing by the number of individuals in that group

Previous reports have identified p.Asn340Ser to be a recurrent variant [5, 22]. The present study has validated and extended this further by identifying 25 (20 unrelated) PCDH19-GCE cases (Table 1). Another recurrent variant (p.Tyr366Leufs*10) reported in 30 (11 unrelated) PCDH19-GCE cases was also identified (Table 2). Of the 25 patients with a p.Asn340Ser variant, almost half (40.0%, 10/25) had normal or borderline cognitive function, 28.0% (7/25) had mild or moderate ID, with the remaining 32.0% either unclear (6/25) or not reported (2/25; Fig. 2a). Further, psychiatric comorbidities primarily included autistic or hyperactive features, or both, with over half of the cases reported as having no psychiatric comorbidity (55.0%, 11/20). Psychiatric reports were not specified for five cases. Of the 30 patients with a p.Tyr366Leufs*10 variant, just over a quarter (26.7%, 8/30) had normal cognitive function, 23.3% (7/30) had mild or moderate ID, and 26.7% (8/30) had severe or profound ID, with the remaining 23.3% either not reported (5/30) or not specifying the degree of ID (2/30; Fig. 2a). Psychiatric comorbidities were predominantly hyperactive (36.7%, 11/30), with one third of cases reported as having no psychiatric comorbidity (33.3%, 10/30). Psychiatric reports were not specified for three cases.

**Table 1:** PCDH19 Variant: p.Asn340Ser (c.1019A>G)

*#*	Age at study	Seizure onset			Subsequent seizure types	Fever sensitivity^+/−^	Seizure offset	Inheritance	Language delay^+/−^	Intellectual disability	Psychiatric comorbidity	Reference
		Age	Type	Fever^+/−^								
1	3.5y	9m	GTC	+	F, SE	+	Controlled	*De novo*	+	Mild	None	[4]
2	6y	8m	F	NS	GTC	+	Ongoing	*De novo*	+	Mild	BD	[4]
3	44y	8m	TC	NS	−	NS	17y	Unknown	NS	Normal	NS	[8]^a^
4	16y	6m	F	+	TC, SE	+	NS	Maternal	NS	Mild	No autistic traits^*^	[8, 54]^a^
5	11y	10m	F	+	−	+	NS	*De novo*	+	Moderate	No autistic traits^*^	[8, 54]
6	7.5y	12m	FBTC	+	−	+	Ongoing	*De novo*	NS	Normal	None	[9]
7	6y	13m	TC	NS	NS	NS	Ongoing	#Maternal	NS	Moderate	Autism	[11]^b^
8	3y	17m	TC	NS	NS	NS	NS	#Maternal	+	NS	NS	[11]^b^
9	NS	12m	NS	NS	NS	NS	14y	*De novo*	NS	Normal	None	[11]^b^
10	5y	13m	F	NS	FBTC	+	Ongoing	*De novo*	NS	Normal	None	[55]^c^
11	5y	25m	GTC	−	None	−	25m	*De novo*	NS	Normal	None	[55]^c^
12	NS	NS	NS	NS	NS	NS	NS	Unknown	NS	NS	NS	[22]
13	32y	15m	NS	−	F, GTC, SE	−	NS	Unknown	NS	Mild	ASD	[56]
14	5.5y	10m	F	NS	−	NS	NS	*De novo*	NS	Borderline	Psychosis, ED, no autistic traits^*^	[54]
15	10y	5m	F	NS	−	NS	NS	*De novo*	NS	DQ 72 (NS)	Autistic traits^*^	[54]
16	8y	12m	F	NS	−	NS	NS	#Maternal	NS	Normal	No autistic traits^*^	[54]
17	8y	11m	C	+	NS	+	5.5y	#Maternal	−	Normal	None	[23]^d^
18	NS	N/A	N/A	N/A	N/A	N/A	N/A	NS	−	Normal	None	[23]^d^
19	6y	15m	HC	+	TC, F, T, SE	+	Ongoing	Unknown	NS	Moderate	Autistic, hyperactive	[16]
20	8y	5m	F, T	+	−	+	Ongoing	*De novo*	NS	DQ 44 (7y4m)	NS	[57]
21	5.5y	8m	TC, F	+	−	+	Ongoing	Maternal	NS	DQ 38 (4y7m)	Autistic, hyperactive	[57]
22	NS	NS	NS	+	NS	+	NS	*De novo*	NS	NS	NS	[5]^e^
23	9y	7m	GTC	NS	F	+	Ongoing	*De novo*	NS	Yes	Autism, aggression	[24]
24	3y	8m	GTC	NS	F, MC	+	Ongoing	Maternal	NS	Yes	Autism, aggression	[24]
25	7y	18m	GTC	NS	−	+	Ongoing	*De novo*	NS	Normal	None	[24]

*GTC* generalized tonic-clonic seizure, *F* focal seizure, *SE* status epilepticus, *BD* behavioral disturbances, *NS* not specified, *TC* tonic-clonic seizure, *FBTC* focal-to-bilateral seizure, *ASD* autism spectrum disorder, *MC* myoclonic seizure, *ED* eating disorder, *C* clonic seizure, *N/A* not applicable, *HC* hemiclonic seizure, *T* tonic seizure

^*^Information obtained from the author

#Asymptomatic female

^abcd^Familial relationships (^a^mother/daughter; ^b^mother/daughters; ^c^twins; ^d^mother/daughter)

^e^Reported as p.Asn370Ser

**Table 2:** PCDH19 Variant: p.Tyr366Leufs*10 (c.1091dupC or c.1091_1092insC)

*#*	Age at study	Seizure onset			Subsequent seizure types	Fever sensitivity^+/−^	Seizure offset	Inheritance	Language delay^+/−^	Intellectual disability	Psychiatric comorbidity	Reference
		Age	Type	Fever^+/−^								
1	23y	18m	TC	NS	NS	NS	NS	Paternal	NS	Profound	Hyperactive	[1, 2, 27]^a^
2	22y	NS	NS	NS	NS	NS	NS	Paternal	NS	Normal	None	[1, 2, 27]^a^
3	21y	NS	NS	NS	NS	NS	NS	Paternal	NS	Normal	None	[1, 2, 27]^a^
4	20y	NS	NS	NS	NS	NS	NS	Paternal	+	Mild	Hyperactive	[1, 2, 27]^a^
5	22y	NS	NS	NS	NS	NS	NS	Paternal	NS	Normal	None	[1, 2, 27]^a^
6	18y	NS	NS	NS	NS	NS	NS	Paternal	NS	Normal	None	[1, 2, 27]^a^
7	8y	NS	NS	NS	NS	NS	NS	Paternal	NS	Normal	None	[1, 2, 27]^a^
8	14y	NS	NS	NS	NS	NS	NS	Paternal	NS	Severe	Hyperactive	[1, 2, 27]^a^
9	12y	NS	F	NS	A, GTC	NS	NS	Paternal	NS	Normal	None	[1, 2, 27]^a^
10	11y	NS	NS	NS	NS	NS	NS	Paternal	NS	Normal	None	[1, 2, 27]^a^
11	14y	NS	NS	NS	F, GTC	NS	NS	Paternal	NS	Profound	Hyperactive	[1, 2, 27]^a^
12	8y	NS	NS	NS	NS	NS	NS	Paternal	NS	Moderate	Hyperactive	[1, 2, 27]^a^
13	6y	NS	NS	NS	NS	NS	NS	Paternal	NS	Moderate	Hyperactive	[1, 2, 27]^a^
14	6y	NS	NS	NS	NS	NS	NS	Paternal	NS	Severe	Hyperactive	[1, 2, 27]^a^
15	5y	NS	NS	NS	NS	NS	NS	Paternal	NS	Severe	Hyperactive	[1, 2, 27]^a^
16	2y	NS	NS	NS	NS	NS	NS	Maternal	NS	Mild	None	[1, 2]^a^
17	2y	NS	NS	NS	NS	NS	NS	Maternal	NS	NS	None	[1, 2]^a^
18	≥5y	4m	GTC	−	A, F, SE	NS	Ongoing	Maternal	+	Severe	BD, hyperactive, impulsive	[2, 27, 58]^a^
19	NS	7m	NS	NS	NS	NS	NS	Maternal	NS	NS	NS	[2, 27, 58]^a^
20	NS	14m	NS	+	NS	NS	NS	Paternal	NS	NS	NS	[2, 27, 58]^a^
21	14y	7m	GTC	NS	−	+	11y	Paternal	+	Severe	Hyperactive	[55]^a^
22	NS	NS	NS	NS	NS	NS	NS	*De novo*	NS	NS	NS	[22]
23	7.5y	17m	F	NS	−	NS	NS	*De novo*	NS	Mild	Autistic traits^*^	[54]
24	9y	2m	F	NS	−	NS	NS	*De novo*	NS	NS	No ASD^*^	[54]
25	3y	6m	FBTC, F	NS	−	−	Ongoing	*De novo*	+	Normal	AD, hyperactive, No ASD^*^	[49, 54]
26	13y	17m	TC	−	−	+	12y	*De novo*	NS	Moderate	Autistic traits	[16]
27	8y	5m	F	+	T	+	Ongoing	Unknown	NS	Mild	Impulsive	[57]^b^
28	1y	6m	F	NS	GTC	+	Ongoing	*De novo*	NS	Yes	None	[24]
29	4y	11m	GTC, F	NS	F	+	Ongoing	*De novo*	NS	Yes	AD	[24]
30	7y	7m	NS	NS	GTC, SE	+	NS	Unknown	NS	Severe	Autistic traits^*^	[59]

*FBTC* Focal-to-bilateral seizure, *F* focal seizure, *NS* not specified, *AD* attention-deficit, *GTC* generalized tonic-clonic seizure, A absence seizure, *DB* destructive behavior, *SE* status epilepticus, *T* tonic seizure, *TC* tonic-clonic seizure

^*^Information obtained from author

^a^Original EFMR family

^b^Reported as c.1300_1301insC

### Neuropsychiatric profile

#### Cognitive function

The cognitive profile associated with PCDH19-GCE (*n* = 195) was found to be highly heterogenous, ranging from normal cognitive function (28.2%) to borderline (5.1%), mild (27.2%), moderate (22.1%), or severe to profound (17.4%) ID (see Supplementary Table 6). We observed that development prior to the onset of seizures was reported to be delayed in approximately 15% of the cases.

#### Psychiatric comorbidities

Of the 213 cases where psychiatric information was provided, autistic features were most prominent (19.7%), followed by hyperactive and/or attention-deficit (11.7%), and behavioral disturbances (6.1%). Many reports described individuals with multiple psychiatric comorbidities (21.6%) that predominantly included combinations of autistic, aggressive, hyperactive, and/or obsessive features (see Supplementary raw data for a complete list).

### Genotype–phenotype association

To determine whether age at seizure onset, mutation type, variant location, or mode of inheritance were associated with cognitive function, a linear regression was performed. Age at the time of the study was also included in the model as a covariate to control any confounding effects that age may have had on the severity of reported ID [25]. Of all the variables tested in the model, only age at seizure onset was significantly associated with cognitive function (*p* *=* 4.127 × 10^−7^; Fig. 2b). Specifically, individuals with an early seizure onset had an average ID severity that was 1.3 units greater than individuals with a late seizure onset, holding other predictors in the model constant (estimate = 1.30, 95% CI: 0.80, 1.80; Table 3). A factorial ANOVA was then performed to ascertain whether the mutation type or the variant location were associated with seizure onset. No significant associations were found (Table 4).

**Table 3:** Estimated marginal means (controlling for covariates)

Age at seizure onset	Mean ID severity	Standard error
≤12 months (“early”)	2.2	0.1
>12 months (“late”)	0.9	0.2

NB: scale: (0) “normal”, (1) “borderline”, (2) “mild ID”, (3) “moderate ID”, or (4) “severe/profound ID”

**Table 4:** Non-significant associations

	Psychiatric comorbidity	Age at seizure onset
Mutation type		
Truncating versus missense	*χ*^2^_(1, *n* = 210)_ = 0.46, *p* *=* 0.497	*F*_(195)_ = 0.41, *p* = 0.523
Variant Location		
Early versus late	*χ*^2^_(1, *n* = 216)_ = 0.08, *p* *=* 0.778	*F*_(195)_ = 0.49, *p* = 0.487
Inheritance		
Sporadic versus familial	*χ*^2^_(1, *n* = 193)_ = 0.75, *p* *=* 0.385	

### Non-significant associations

To determine whether any predictor variable was associated with the presence of a psychiatric comorbidity, a series of Pearson’s chi-squared tests of independence were performed. There was a trend toward earlier seizure onset being associated with the presence of a psychiatric comorbidity, *χ*^2^_(1, *n* = 205)_ = 3.01, *p* *=* .083, however, this was not statistically significant. All other tested associations were non-significant (Table 4).

### PCDH19 mosaic males

There have been ten reported cases of *PCDH19* mutations causing a GCE-like phenotype in males. *PCDH19* mutations were initially thought to only affect females, however, in 2009, Depienne and colleagues [4] described a *SCN1A*-negative male diagnosed with “Dravet syndrome”, as having a *de novo* deletion on chromosome Xq22.1 that spanned the entire *PCDH19* gene. Using fluorescence *in situ* hybridization, the mosaic status of this *PCDH19*-variant male was confirmed, with a “normal” *PCDH19* allele detected in 53% of the skin fibroblasts. A second case was described by Thiffault et al. (2016) [6]. Sanger sequencing revealed an exon 1 protein-truncating variant in a mosaic status that was associated with focal myoclonic, as well as tonic-clonic seizures, at the age of 9 months. Two additional mosaic males were recently reported by Terracciano et al. (2016) [5]. The first, a 4-year-old boy, presented with an afebrile hypotonic seizure at the age of 9 months. The second, a 3.5-year-old boy, presented with a 24-h cluster of febrile seizures at the age of 10 months. Multigene panel revealed an exon 1 nonsense and missense substitution in each case, respectively. Recently, a male mosaic for a *PCDH19* missense mutation believed to affect the canonical splice donor site in the first intron (c.2147+2T>C) was reported [26], and, subsequently, an additional five mosaic males have been identified [17]. All six males exhibited a clinical profile corresponding to the female phenotype. Nine of the ten reported *PCDH19* mosaic males have been described as having comorbid psychiatric features [4–6, 17, 26]. For example, the case described by Thiffault and colleagues [6] involved a young boy with behavioral disturbances (i.e., aggression and rigidity) that became evident by the age of 3 years. At the time of the study, he had been diagnosed with attention-deficit hyperactivity disorder (ADHD), anxiety, obsessive-compulsive disorder (OCD), and oppositional defiant disorder. All cases of affected males with a normal complement of sex chromosomes have arisen *de novo*, suggesting that a somatic *PCDH19* mutation during early development resulted in a mixed population of *PCDH19* mutant and wild-type cells, and therefore cellular expression resembling that of an affected female.

### PCDH19 transmitting males

It is generally considered that hemizygous or “transmitting” males are unaffected or asymptomatic [1, 2, 27]. While epilepsy has not been reported in these males, there is some evidence to suggest that there is a mild phenotype associated with this transmitting status in males. The first indication of such arose from the observations of Scheffer et al. in 2008 [3]. In this study, five males were all described as inflexible, having rigid and controlling personalities, and obsessive interests and traits (e.g., obsessively repeating details in conversation). Such characteristics are particularly common in ASD (e.g., inflexibility) and OCD (e.g., repetitive behaviors). In addition, some transmitting fathers of the affected daughters have varying degrees of ID [4, 16]. Lastly, *PCDH19* mutations have been reported in a male with autism [28], a male with Asperger’s syndrome [16], and two males with ID [29]. These reports suggest that a psychiatric profile may be evident in some transmitting males, and that *PCDH19* is involved in other neurodevelopmental disorders.

## Discussion

This review is the first to systematically characterize the reported neuropsychiatric profile of PCDH19-GCE, and examine any associations between clinical and molecular factors, and neuropsychiatric outcomes. We have demonstrated that an earlier seizure onset is significantly associated with more severe ID. We have also shown that there is no association between the type  or location of a *PCDH19* mutation and seizure onset, and confirmed that onset is often precipitated by fever. Although the association of early seizure onset with more severe ID may simply reflect the underlying severity of the disorder in that individual, it is also possible that the early seizure activity may be contributing to adverse cognitive and behavioral outcomes. There are “critical periods” of development during which the brain undergoes changes that are crucial to the formation of certain behaviors and various cognitive processes [30, 31]. Functional changes in the frontal cortical brain regions, in particular, coincide with cognitive and behavioral alterations known to occur during early development [30, 32]. We have observed that a majority of first seizures in PCDH19-GCE occur at a median age of 10 months. It is at this time that the frontal cortex shows an increase in glucose metabolism [33], with total brain volume increasing by 101% in the first year of life [34]. There is also a rapid elaboration of new synapses in the first 2 years of life that corresponds to an increase in cortical gray matter [35, 36]. The frontal cortex is involved in a diverse range of functions that can be broadly referred to as “cognition” [37]. Injury to this region has been associated with deficits in executive functioning (i.e., attention), as well as psychiatric conditions including schizophrenia, depression, and OCD [37]. It is therefore reasonable to speculate that seizure activity within the first 12 months of life may be more likely to disrupt the neural development and lead to cognitive dysfunction.

Given the clinical similarities involving age at seizure onset and fever sensitivity shared by PCDH19-GCE and Dravet syndrome, we investigated whether a similar association between ID severity and age at seizure onset has been demonstrated in the Dravet literature. Brunklaus et al. (2012) demonstrated an association between early focal seizures with impaired awareness ≤24 months (yes/no) and worse developmental outcome [25]. Patients with Dravet syndrome with the highest seizure burden were reported to also suffer from more comorbidities [38]. Further, early seizure onset has been associated with Dravet sydrome rather than GEFS+ in children with *SCN1A* mutations [39]. Importantly, we recently defined a new profound *SCN1A* developmental and epileptic encephalopathy far more severe than Dravet syndrome that is associated with an even earlier (6–12 weeks) seizure onset [40].

Considering this question from a different perspective, McIntosh et al. (2010) investigated whether seizure onset in Dravet syndrome triggered by vaccination (called vaccination proximate) had a more severe clinical outcome than patients whose seizure onset was not related to vaccination (vaccination distal). While the two groups did not differ in severity of cognitive decline, they differed significantly in the average age at seizure onset, with onset being earlier by approximately 8 weeks in the vaccination proximate group [41]. As there are anecdotal reports of vaccination triggering seizures in PCDH19-GCE, it would be interesting to ascertain whether PCDH19-GCE demonstrates a similar association between vaccination and age at seizure onset.

We also identified two recurrent variants p.Asn340Ser and p.Tyr366Leufs*10. Both recurrent variants were found at a similar location within the *PCDH19* gene, suggesting that this region may be vulnerable to or selected for genetic mutation. These recurrent variants provided some scope for determining a genotype–phenotype association. We were able to utilize the recurrence of these two variants to demonstrate, for the first time, that there is no association between these specific PCDH19 variants and the type and/or severity of symptoms, at least at a qualitative level. However, it is feasible to postulate that a milder phenotype may be associated with the p.Asn340Ser variant. The identification of additional recurrent cases is required to validate these findings and draw a more definitive conclusion. Heterogeneity was typically observed among related individuals, suggesting that other mechanisms such as hormones [42, 43], X-chromosome inactivation [44], or other genetic or environmental factors may be the underlying explanation for the variable clinical expressivity associated with PCDH19-GCE. One interesting finding that emerged was the absence of any paternally inherited p.Asn340Ser variants. Complete pedigree information regarding these cases is required to determine whether this is, in fact, a true observation. In addition, the annotation of p.Tyr366Leufs*10 varied in the literature. As such, there may have been additional variants that have been reported across multiple individuals and families that have not been correctly identified. Such additional recurrent cases will allow for more detailed quantitative analyses.

This review has revealed that the neuropsychiatric profile of PCDH19-GCE varies considerably across individuals and within families. Current reports concerning psychiatric comorbidities in PCDH19-GCE are incomplete. This review provides some insight into the type of psychiatric comorbidities that likely exist in association with *PCDH19* mutations. In line with previous reports [11, 14, 45], we observe that autistic features are most prominent. A novel finding to emerge is that hyperactivity is frequently observed. This finding is reflected in a recent animal model study showing that heterozygous female *Pcdh19* knockout mice show hyperactivity in social interactions, under stress and with advancing age [46]. Overall, the features associated with ADHD, ASD, and OCD are observed in PCDH19-GCE at rates much higher than those observed in the general population [47]. Although these rates are comparable to those reported among individuals with ID [48], 25% of reviewed cases have normal cognitive function in association with psychiatric comorbidities. These results should be considered formative due to limited data, specifically targeting the presence and/or severity of psychiatric symptomatology. As over 60% of reviewed cases are associated with some form of psychiatric comorbidity, a comprehensive and standardized assessment of the psychiatric profile associated with *PCDH19* mutations is warranted [49].

There were no reported psychiatric comorbidities in over 75% of individuals with normal cognitive function. Future research is recommended to determine what factors are unique to this group, as this might explain what causes the clinical variability observed in PCDH19-GCE. Previous reports suggested that ID became apparent sometime after seizure onset [8, 9, 14], suggesting that seizure and epileptic activity may have contributed to the cognitive deficits. However, we observed that development prior to the onset of seizures was delayed for 15% of individual,s indicating that *PCDH19* mutations produced a developmental encephalopathy, as well as an epileptic encephalopathy in some cases. Given that prior development was not reported, unclear, or unknown in 130 cases, and that obtaining such information retrospectively or prior to the onset of seizures can be challenging, previous reports were likely underestimating the proportion of individuals showing signs of delay prior to seizure onset. It was also noted that dysfunction specific to executive functioning was reported, such as problems with planning and organization [50], abstract reasoning [45], or lack of inhibitory control [49]. Therefore, executive functioning may be compromised in PCDH19-GCE. Moreover, definitions of ID now include deficits in adaptive behavior, with the severity of ID based on the adaptive behavior impairment, rather than exclusively on the IQ score [47].

## Conclusion

Given the limited information in the literature concerning comorbid symptomatology, there is a need to formally characterize the neuropsychiatric profile of PCDH19-GCE. Neuropsychiatric disorders can be very responsive to early intervention; [51–53] therefore, a better understanding of these comorbidities may help to inform treatment and ultimately lead to better developmental outcomes for individuals affected by PCDH19-GCE. In addition, transmitting males may exhibit mild neuropsychiatric features. An assessment of these males may identify a clinical profile unique to this group, which may lead to carrier testing and has implications for genetic counseling. We have shown that seizure onset within the first 12 months is significantly associated with more severe ID. Therefore, knowledge of an individual’s seizure onset will aid prognostic counseling, providing valuable information for clinicians managing affected individuals and their families.

## Electronic supplementary material

Dataset

Supplementary Material

Supplementary Material
