# Supplementary material for: A systematic review and meta-analysis of 271 PCDH19-variant individuals identifies psychiatric comorbidities, and association of seizure onset and disease severity
Source: Mol Psychiatry. 2018 Jun 11;24(2):241–51. doi: 10.1038/s41380-018-0066-9 (PMC6344372; doi:10.1038/s41380-018-0066-9)

**Supplementary Material**

**Table 1.** Seizure onset precipitate

|  | | Frequency | Percent |
| --- | --- | --- | --- |
| Valid | Not applicable | 3 | 1.1 |
|  | Afebrile | 16 | 5.9 |
|  | Fever | 86 | 31.7 |
|  | Not reported | 49 | 18.1 |
|  | Unclear | 111 | 41.0 |
|  | Unknown | 2 | .7 |
|  | Vaccination | 4 | 1.5 |
|  | Total | 271 | 100.0 |
| Missing |  | 0 |  |
| Total | | 271 |  |

**Table 2.** Variant location

|  | | Frequency | Percent |
| --- | --- | --- | --- |
| Valid | EC1 | 32 | 11.7 |
|  | EC2 | 38 | 13.9 |
|  | EC3 | 55 | 20.1 |
|  | EC4 | 63 | 24.8 |
|  | EC5 | 17 | 6.2 |
|  | EC5-EC6 | 1 | .4 |
|  | EC6 | 29 | 10.6 |
|  | Cytoplasmic | 20 | 7.3 |
|  | Not applicable | 16 | 6.2 |
|  | Total | 271 | 100.0 |
| Missing |  | 0 |  |
| Total | | 271 |  |

**Table 3.** Type of mutation

|  | | Frequency | Percent |
| --- | --- | --- | --- |
| Valid | Frameshift | 74 | 27.3 |
|  | In-frame deletion | 1 | .4 |
|  | In-frame duplication | 3 | 1.1 |
|  | In-frame insertion | 1 | .4 |
|  | Missense substitution | 122 | 45.0 |
|  | Nonsense substitution | 52 | 19.2 |
|  | Nonsense; Missense | 1 | .4 |
|  | Silent substitution | 1 | .4 |
|  | Splice-site | 5 | 1.8 |
|  | Whole/partial gene deletion | 11 | 4.0 |
|  | Total | 271 | 100.0 |
| Missing |  | 0 |  |
| Total | | 271 |  |

**Table 4.** PCDH19 cDNA

| **cDNA** | **Protein** | **Correct Annotation** | **Frequency** | **cDNA** | **Protein** | **Correct Annotation** | **Frequency** |
| --- | --- | --- | --- | --- | --- | --- | --- |
| 1017delC | N340Mfs*28 |  | 1 | 1522_1528delATCAATC | I508Pfs*59 |  | 1 |
| 1019A>G (17 recurrent) | N340S |  | 25 | 152dupT | A51Rfs*37 |  | 1 |
| 1022A>G | D341G |  | 1 | 1537G>C | G513R |  | 1 |
| 1023C>G | D341E |  | 1 | 1615G>C | G539R |  | 1 |
| 1026_1027delinsAA | N342_P343delinsKT |  | 1 | 1628T>C | L543P |  | 1 |
| 1031C>G (unrel.) | P344R |  | 2 | 1649G>A | R550P | R550Q | 1 |
| 1031C>T | P344L |  | 1 | 1671C>G (sibs) | N557K |  | 2 |
| 1036_1040dup | N347Kfs*23 |  | 1 | 1681G>A | P561S | 1681C>T | 1 |
| 1048C>G | S350* | 1049C>G | 1 | 1682C>G (sibs+unrel.) | P561R |  | 3 |
| 1091_1092insC (recurrent*) | Y366Lfs*10 |  | 2 | 1700C>T (unrel.) | P567L |  | 2 |
| 1091delC (DZ twins+unrel.) | P364fs | P364Rfs*4 | 3 | 1765_1766delTG | V589Cfs*8 | 1765_1766delGT | 1 |
| 1091dupC (9 recurrent*) | Y366Lfs*10 |  | 28 | 1780G>C | D594H |  | 1 |
| 1098C>G | Y366* |  | 1 | 1786G>C | D596H |  | 1 |
| 1123G>T | D375Y |  | 1 | 1787A>T | D596V |  | 1 |
| 1129G>A | D377N |  | 1 | 1802G>A | G601D |  | 1 |
| 1129G>C | D377H |  | 1 | 1804C>T (unrel.) | R602* |  | 2 |
| 1131C>A | D377E |  | 1 | 1825G>T | E609* |  | 1 |
| 1143dupT | G381Wfs*19 |  | 1 | 1852G>A | D618N |  | 1 |
| 1178C>T | P393L |  | 1 | 1863dupT | G622Wfs*18 |  | 1 |
| 1183C>T | R395* |  | 1 | 1864G>C | G622R |  | 1 |
| 1184G>C | R395P |  | 1 | 1924G>A | V642M |  | 1 |
| 1192G>T | E398* |  | 1 | 1955T>C | L652P |  | 1 |
| 1211C>T | T404I |  | 1 | 1956_1959delCTCT | S653Pfs*6 |  | 1 |
| 1240G>C | E414Q |  | 1 | 2012C>G (rel.+1 unrel.) | S671* |  | 7 |
| 1240G>A | E414K |  | 1 | 2019delC | S674Lfs*2 |  | 1 |
| 1298T>C | L433P |  | 1 | 2030_2031insT (rel.) | L677fs*717 | L677Ffs*41 | 7 |
| 1300_1301delCA (MZ twins) | Q434Efs*11 |  | 2 | 2147+2T>C | p.? (exon/intron 1) |  | 1 |
| 1322T>A (rel.) | V441E |  | 10 | 2156T>G | L719* |  | 1 |
| 134_135ACdel (sibs) | D45Gfs*43 |  | 2 | 215T>G | V72G |  | 1 |
| 1347_1348insAAC | N449_H450insN |  | 1 | 2341dupA | I781Nfs*3 |  | 1 |
| 1352C>T | P451L |  | 1 | 2359C>T | R787C |  | 1 |
| 1375C>T | Q459* |  | 1 | 241dupC | L81Pfs*8 |  | 1 |
| 1408_1417delGCCTATCTGC | A470Sfs*96 |  | 1 | 242T>G (recurrent) | L81R |  | 4 |
| 142G>T (sibs) | E48* |  | 2 | 253C>T (rel. + 1 unrel.) | Q85* |  | 5 |
| 1456G>C | G486R |  | 1 | 2567delCGGCACT | Not provided | 2567delAGGGGCC, Q856Pfs*6 | 1 |
| 1464_1466delCTC | S489delS |  | 1 | 2568C>T^#^ | S856S | 2563C>T, S855S | 1 |
| 1521dupC | I508Hfs*15 |  | 1 | 2617-1G>A | p.? (intron 3/exon 4) |  | 1 |

NB: *del* = deletion, *DZ* = dizygotic, *dup* = duplication, *ins* = insertion, *sibs* = siblings, *unrel/rel.* = unrelated/related individuals, *same mutation, ~ possible repeated case, ^#^unlikely to be clinically relevant

| **cDNA** | **Protein** | **Correct Annotation** | **Frequency** | **cDNA** | **Protein** | **Correct Annotation** | **Frequency** |  |
| --- | --- | --- | --- | --- | --- | --- | --- | --- |
| 2631_2634delTTTT | F878Tfs*5 |  | 1 | 617T>A | F206Y |  | 1 |  |
| 2656C>T (5 recurrent) | R886* |  | 8 | 695A>G (recurrent) | N232S |  | 4 |  |
| 2675-6A>G | p.? (intron 4/exon 5) |  | 1 | 697_700delinsTAAC (sibs) | D233* |  | 2 |  |
| 2675+1G>C (unrel.) | p.? (exon/intron 4) |  | 2 | 701A>G | N234S |  | 1 |  |
| 2697dupA | E900Rfs*8 |  | 1 | 706C>T | P236S |  | 1 |  |
| 269A>T | D90V |  | 1 | 718G>T | E240* |  | 1 |  |
| 2705dupA (rel.) | D902Kfs*6 | N902Kfs*6 | 2 | 729C>A | Y243* |  | 1 |  |
| 2873C>T | R958Q | 2873G>A | 1 | 730dupG | A244Gfs*76 |  | 1 | |
| 2903dupA | D968Efs*18 |  | 1 | 746A>G | E249G |  | 1 |  |
| 2926G>A^#^ | D976N | 3070G>A, D1024N | 1 | 747A>T | E249D |  | 1 |  |
| 339C>A | C113* |  | 1 | 74T>C (sibs) | L25P |  | 2 |  |
| 352G>T | E118* |  | 2~ | 772_773delAT | I258Pfs*61 |  | 1 |  |
| 357delC (unrel.) | K120Rfs*3 |  | 2 | 785C>A | A262D |  | 1 |  |
| 361G>A | D121N |  | 1 | 78delG | K26Nfs*4 |  | 1 |  |
| 370G>A (sibs) | D124N |  | 2 | 790G>C (unrel.) | D264H |  | 2 |  |
| 415_423dup (rel.) | S139_A141dup |  | 3 | 799G>T | E267* |  | 1 |  |
| 416C>A | S139* |  | 1 | 823T>A | Y275N |  | 1 |  |
| 416C>T | S139L |  | 1 | 824A>C | Y275S |  | 1 |  |
| 424delG | A142Pfs*70 |  | 1 | 826T>C | S276P |  | 1 |  |
| 437C>G (rel.) | T146R |  | 3 | 83C>A | S28* |  | 1 |  |
| 445C>T | P149S |  | 1 | 83C>A & 90A>G | S28* & E90E |  | 1 |  |
| 457G>A | A153T |  | 1 | 840C>G | Y280* |  | 1 |  |
| 462C>A | Y154* |  | 1 | 859G>T | E287* |  | 1 |  |
| 462C>G | Y154* |  | 1 | 859G>T & 3319C>G | E287* & R1107G |  | 1 |  |
| 469G>A | D157N |  | 1 | 918C>G | Y306* |  | 1 |  |
| 471C>A | D157E |  | 1 | 919G>A | E313K | E307K | 1 |  |
| 473C>G | S158* |  | 1 | 91G>A | E31K |  | 1 |  |
| 488T>G | V163G |  | 1 | 937G>A | E307K | E313K | 1 |  |
| 497_498insA | Y166* |  | 1 | 949C>T | Q317* |  | 1 |  |
| 506delC (unrel.) | T169Sfs*43 |  | 2 | 958dupG | D320Gfs*22 |  | 1 |  |
| 514dupG | E172Gfs*54 |  | 1 | 964G>C | G322R |  | 1 |  |
| 569T>G | L190R |  | 1 | C>G | H146Q | 1935C>G, H645Q | 1 |  |
| 571G>C | V191L |  | 1 | T>C | V91A | 1770T>C, V590A | 1 |  |
| 593G>T | R198L |  | 1 | **Total** |  |  | **145** |  |
| 595G>C | E199Q |  | 1 |  |  |  |  |  |
| 605C>A | S202* |  | 1 | Partial gene deletion |  |  | 1 |  |
| 608A>C & 617T>G | H203P & F206C |  | 1 | Whole gene deletion |  |  | 10 |  |

NB: *del* = deletion, *DZ* = dizygotic, *dup* = duplication, *ins* = insertion, *sibs* = siblings, *unrel/rel.* = unrelated/related individuals, *same mutation, ~ possible repeated case, ^#^unlikely to be clinically relevant

All corrections based on NM_001184880.1

**Table 5.** Inheritance

|  | | Frequency | Percent |
| --- | --- | --- | --- |
| Valid | #Maternal | 9 | 3.3 |
|  | *De novo* | 110 | 40.6 |
|  | *De novo*; Paternal | 1 | .4 |
|  | Familial | 3 | 1.1 |
|  | Maternal | 32 | 11.8 |
|  | Not the father | 1 | .4 |
|  | Not the mother | 2 | .7 |
|  | Not reported | 10 | 3.7 |
|  | Paternal | 64 | 23.6 |
|  | Unknown | 39 | 14.4 |
|  | Total | 271 | 100.0 |
| Missing |  | 0 |  |
| Total | | 271 |  |

**Table 6.**  Cognitive function

|  | | Frequency | Percent |
| --- | --- | --- | --- |
| Valid | Normal | 55 | 28.2 |
|  | Borderline | 10 | 5.1 |
|  | Mild ID | 53 | 27.2 |
|  | Moderate ID | 43 | 22.1 |
|  | Severe/Profound ID | 34 | 17.4 |
|  | Total | 195 | 100.0 |
| Missing |  | 76 |  |
| Total | | 271 |  |

**Figure 1.**  Distribution of age at seizure onset for males and females


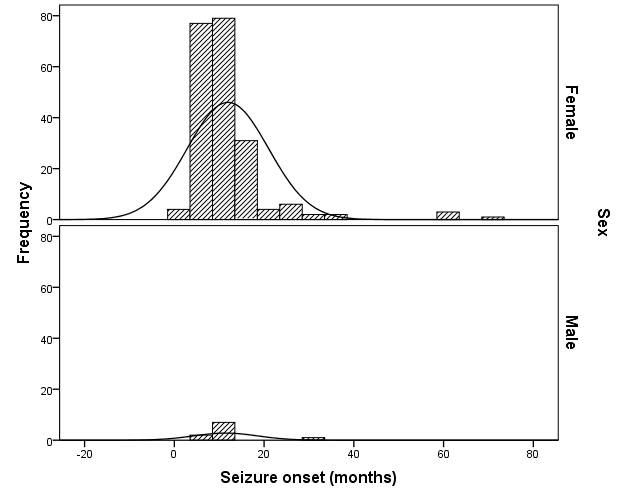

Supplement: Supplementary file 2 — Supplementary Material [file 41380_2018_66_MOESM2_ESM.docx]
